# Endogenous Type I CRISPR-Cas: From Foreign DNA Defense to Prokaryotic Engineering

**DOI:** 10.3389/fbioe.2020.00062

**Published:** 2020-03-04

**Authors:** Yanli Zheng, Jie Li, Baiyang Wang, Jiamei Han, Yile Hao, Shengchen Wang, Xiangdong Ma, Shihui Yang, Lixin Ma, Li Yi, Wenfang Peng

**Affiliations:** State Key Laboratory of Biocatalysis and Enzyme Engineering, Hubei Collaborative Innovation Center for Green Transformation of Bio-resources, Hubei Key Laboratory of Industrial Biotechnology, School of Life Sciences, Hubei University, Wuhan, China

**Keywords:** endogenous type I CRISPR-Cas systems, mechanisms of action, DNA targeting, genome editing, selective killing, antimicrobials, gene expression modulation

## Abstract

Establishment of production platforms through prokaryotic engineering in microbial organisms would be one of the most efficient means for chemicals, protein, and biofuels production. Despite the fact that CRISPR (clustered regularly interspaced short palindromic repeats)–based technologies have readily emerged as powerful and versatile tools for genetic manipulations, their applications are generally limited in prokaryotes, possibly owing to the large size and severe cytotoxicity of the heterogeneous Cas (CRISPR-associated) effector. Nevertheless, the rich natural occurrence of CRISPR-Cas systems in many bacteria and most archaea holds great potential for endogenous CRISPR-based prokaryotic engineering. The endogenous CRISPR-Cas systems, with type I systems that constitute the most abundant and diverse group, would be repurposed as genetic manipulation tools once they are identified and characterized as functional in their native hosts. This article reviews the major progress made in understanding the mechanisms of invading DNA immunity by type I CRISPR-Cas and summarizes the practical applications of endogenous type I CRISPR-based toolkits for prokaryotic engineering.

## Introduction

Throughout the past billion years, bacteria and archaea have evolved a range of defense mechanisms to defend themselves against their viral predators (Doron et al., 2018), including restriction–modification systems, abortive infections and phage adsorption blocks, and the recently discovered CRISPR-Cas (clustered regularly interspaced short palindromic repeats-CRISPR-associated) systems (Jansen et al., 2002). Unique among these mechanisms, CRISPR immunity functions by storing records of previous invasions to provide immunological memory for a rapid and robust response upon subsequent viral infections.

CRISPR-Cas systems consist of two genetic components, the CRISPR array and *cas* genes encoding Cas proteins. The CRISPR array, featuring the CRISPR-Cas systems, is composed of conserved direct repeats, which are separated by unique sequences derived from the invasive mobile genetic elements (termed as *spacers*) (Bolotin et al., 2005; Mojica et al., 2005; Pourcel et al., 2005). Generally, the CRISPR immunity is driven by the Cas proteins in three distinct molecular stages. The first stage is termed *spacer acquisition*, in which a short DNA stretch is captured from an invading genetic element and incorporated into a CRISPR array as the first spacer immediately after a leader sequence. Then, in the process of *crRNA (CRISPR RNA) biogenesis*, the entire CRISPR array is transcribed into a precursor CRISPR RNA (pre-crRNA) molecule driven by promoter elements embedded in the leader sequence. Following the transcription, cleavage within the repeats of the pre-crRNA by ribonucleases gives mature crRNAs, with each carrying a unique foreign sequence. In the final stage of *target interference*, each crRNA forms a ribonucleoprotein effector complex with Cas proteins and guides the effector machinery to the matching region of the invader through base pairing for destruction (Barrangou et al., 2007; Brouns et al., 2008; Marraffini and Sontheimer, 2008; Garneau et al., 2010).

**FIGURE 1:**
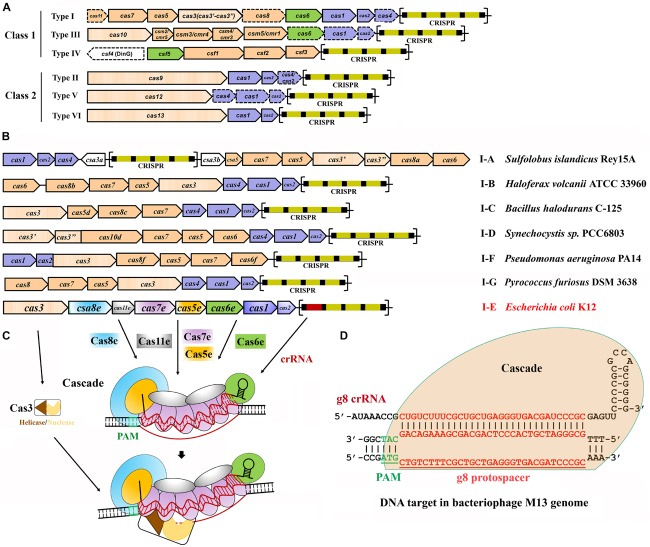
Classification and architecture of CRISPR-Cas systems and interference by type I systems. **(A)** CRISPR-Cas systems are greatly diverse and can be classified into two classes, class 1 and class 2. Class 1 systems encode multisubunit effector complexes; class 2 systems encode single-subunit effectors. Genes that may miss in certain subtypes are indicated with dashed outlines. Genes encoding the components of each interference complex are colored in orange, and those involved in crRNA processing and new spacer acquisition are in green and blue, respectively. The effector nucleases for each subtype are shown with filled vertical lines. CRISPR arrays are indicated, with squares and rectangles representing repeats and spacers, respectively. **(B)** Organization of the CRISPR-Cas loci for the typical type I subtypes. Representative operons for each type are shown, and gene names are indicated. Gene functions are marked with colors the same as shown in **(A)**, except for the subtype I-E of *Escherichia coli* K12. CRISPR arrays are indicated, with squares and rectangles representing repeats and spacers, respectively. **(C)** Schematic of DNA targeting by the representative type I-E of *E. coli* K12. It is composed of a crRNA bound by Cas5 and Cas6 at either end and Cas7 subunits along the guide region, a large subunit (Cas8), and sometimes a small subunit (Cas11). Upon PAM recognition by Cas8, Cascade binding to the target DNA leads to DNA duplex destabilization, allowing crRNA invasion to form a full R-loop. Cas3 is recruited to the R-loop and nicks the replaced strand of the target DNA within the protospacer. **(D)** Schematic showing type I-E Cascade containing a crRNA (g8 crRNA) targeting a sequence on the genome of bacteriophage M13. Sequences of PAM and protospacer are indicated with underlined green and red fonts, respectively [constructed according to Semenova et al. (2011)].

Despite the general immunity stages for all the characterized CRISPR-Cas systems, the Cas proteins and hence the effector complexes vary widely. According to the assortment of *cas* genes and the complexity of their inference complexes, to date, six main types, types I to VI, of CRISPR-Cas systems have been identified and grouped into two classes, classes 1 and 2 (Makarova et al., 2015, 2018;Table 1). Systems of class 1, including types I, III, and IV, are defined by multi-Cas proteins, whereas those of class 2, including types II, V, and VI, utilize a single effector Cas protein (Figure 1A).

**TABLE 1:** The six types of CRISPR-Cas systems.

**Class**	**Type**	**Subtype**	**Spacer acquisition**	**crRNA biogenesis**	**Interference crRNP**	**Type of nucleic acid targets**
1	I	A-G	Cas1, Cas2, Cas4	Cas6/Cas5d	Cascade	DNA
	III	A-F	Cas1, Cas2	Cas6	Csm/Cmr	DNA/RNA
	IV	A-C	Unknown	Csf5	Csf	DNA
2	II	A-C	Cas1, Cas2, Cas4/Csn2, Cas9	RNase III Cas9	Cas9	DNA
	V	A-I, K	Cas1, Cas2, Cas4	Cas12	Cas12	DNA
	VI	A-D	Cas1, Cas2	Cas13	Cas13	RNA

Because of the simplicity of class 2 systems, in which a single Cas protein is sufficient to execute targets binding and cleavage, their effector machineries are relatively easy to be adopted and have been emerged as powerful tools for genome manipulation applications in both prokaryotic and eukaryotic cells. Type II CRISPR-Cas9, as the first identified class 2 system, has been extensively harnessed for genome editing in a wide range of organism, from bacteria to eukaryotic cells, in the past few years (Jinek et al., 2012; Cong et al., 2013; Malina et al., 2013; Jiang et al., 2014). Subsequently, type V CRISPR-Cas12 was characterized and repurposed for genome editing (Zetsche et al., 2015, 2017; Fonfara et al., 2016). These systems have been also engineered for applications beyond genome editing, for example, gene expression regulation via repression or activation, epigenome editing, *in situ* genomic imaging, large-scale genomic screening, and so on (Chen et al., 2013; Cheng et al., 2013; Perez-Pinera et al., 2013; Qi et al., 2013; Shalem et al., 2014; Takei et al., 2017). Type VI CRISPR-Cas13 is the third type in class 2 system, which was demonstrated and developed as a versatile RNA manipulation tool to be used in RNA interference (RNAi), *in vivo* RNA molecule visualization, and nucleic acid detection (Cox et al., 2017; O’Connell, 2019). Recently, several novel single-stranded DNA (ssDNA)–cleaving CRISPR-Cas14 systems belonging to class 2 were identified (Harrington et al., 2018). It is worth to be mentioned that the targeted non-specific ssDNA cleavage activity of Cas14 enabled the system to perform genotyping (Harrington et al., 2018), while it has only about half size of Cas9/Cpf1, thus representing so far the smallest effector nuclease in a single-Cas effector system.

Undoubtedly, class 2 CRISPR-Cas systems have attracted great attention with their fruitful achievements in genome manipulations. However, the engineered targets exhibited an obvious bias toward eukaryotic cells. One of the reasons could be that, as heterologous large-sized nucleases with intrinsic toxicity, the class 2 Cas effectors are hard to be introduced into prokaryotes, particularly those poorly genetically accessible prokaryotic cells. In one recent study, it failed to yield any colony when introducing Cas9 into *Corynebacterium glutamicum* cells even without a guide RNA (gRNA), suggesting the cytotoxicity of the Cas9 *per se* (Jiang et al., 2017). It was also reported that overexpressing a catalytically dead Cas9 (dCas9) in *Escherichia coli* resulted in abnormal morphology and retarded growth, indicating that the cytotoxicity of Cas9 is not solely caused by DNA cleavage but possibly transient non-specific DNA binding across the genome (Cho et al., 2018). Therefore, using endogenous CRISPR-Cas systems of the host for genome engineering could be an effective way to overcome the restriction (Hidalgo-Cantabrana et al., 2019a). In comparison with the imported class 2 systems, all the protein components of endogenous type I systems are present in the cells, excluding any heterologous nuclease. They naturally produce mature crRNA guides without any heterologous helper, which is with particular convenience to conduct, for example, multiplexed genome editing or simultaneous multiple-gene regulation for metabolic pathway engineering by simply supplying an artificial CRISPR array (Luo et al., 2015; Cheng et al., 2017).

It was reported that class 1 systems, primarily types I and III, with only a few reports added recently of type IV (Crowley et al., 2019; Ozcan et al., 2019; Taylor et al., 2019), are present in more than 90% of sequenced genomes of bacteria and archaea (Makarova et al., 2015). Class 1 endogenous systems are much more abundant than class 2 systems (Grissa et al., 2007) and exist not only in mesophiles but also in extreme thermophiles. This is indicative of a great potential to harness the class 1 endogenous systems for applications across many areas of biology. In fact, using type III system for genome editing has been carried out in *Sulfolobus islandicus* (Li et al., 2016). Because the functions and applications of type III systems have been recently reviewed elsewhere (Liu et al., 2018), we will present here the major progress achieved for type I systems, uncovering mechanisms of action of the type I CRISPR-directed immunity, concerning crRNA processing, effector complex assembly, PAM-directed target recognition, seed sequence–mediated target invasion, and Cas3-executed target cleavage, and briefly discuss their recent practical applications, for example, genome editing, antimicrobials, and gene expression regulation, in their prokaryotic hosts.

## Mechanisms of Invading DNA Defense by Type I CRISPR-Cas

Type I CRISPR-Cas systems contain a Cas3 protein as the defining feature of this type with exception for transposon-encoded type I variants (Peters et al., 2017; Klompe et al., 2019; Makarova et al., 2019; Strecker et al., 2019) and are divided into six subtypes, I-A through I-G, with each harboring a specifying Cas8 homolog (Figure 1B and Table 1; Majumdar et al., 2015; Makarova et al., 2015, 2018; Koonin et al., 2017b). Intensive investigations on model type I CRISPR-Cas systems have revealed molecular mechanisms of multi-Cas CRISPR-based antiviral defense, in respect of crRNA processing, effector complex assembly, PAM recognition and R-loop formation, and Cas3-executed DNA target destruction (Figures 1C,D).

### crRNA Processing

**FIGURE 2:**
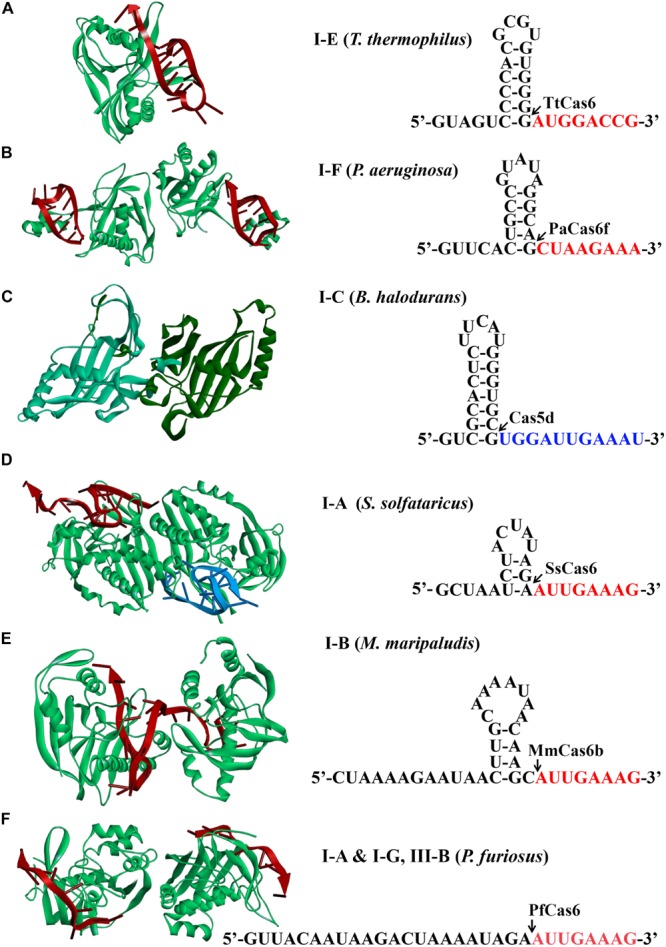
Structures of Cas6 and Cas5d (left) and schematic of CRISPR repeats (right). Structural examples for Cas6 or Cas5d were presented from **(A)** type I-E of *Thermus thermophilus* (TtCas6, PDB code 2Y8W) (Sashital et al., 2011), **(B)** type I-F of *Pseudomonas aeruginosa* (PaCas6, PDB code 2XLK) (Haurwitz et al., 2010), **(C)** type I-C of *Bacillus halodurans* (Cas5d, PDB code 4F3M) (Nam et al., 2012), **(D)** type I-A of *Sulfolobus solfataricus* (SsCas6, PDB code 4ILL) (Shao and Li, 2013), **(E)** type I-B of *Methanococcus maripaludis* (MmCas6, PDB code 4Z7K) (Shao et al., 2016), and **(F)** type I-G of *Pyrococcus furiosus* (PfCas6, PDB code 3PKM) (Wang et al., 2011). The structures of Cas6 are shown in complex with crRNA (red). CRISPR RNAs from type I systems form either stable canonical stem-loop **(A–C)**, mini-stem-loop **(D,E)**, or non–stem-loop **(F)**, structures. The predicted processing sites are indicated by arrows, and sequences are to be present as the 5′ handle of a crRNA is shown in red or blue fonts.

A mature crRNA is an essential element for an active CRISPR-Cas effector complex, in which it functions to guide the recognition of the cognate targets for destruction. It has been reported that biogenesis of mature crRNAs requires cleavage of long CRISPR transcripts within each repeat. In type I systems, an RNA recognition motif (RRM)–containing protein, either a Cas5d for type I-C (Garside et al., 2012; Nam et al., 2012; Punetha et al., 2014) or a Cas6 homolog for rest type I systems (Przybilski et al., 2011; Sashital et al., 2011; Li et al., 2013; Sokolowski et al., 2014; Majumdar et al., 2015; Taylor et al., 2019), recognizes and catalyzes the crRNA maturation. It should be noted that some type III systems also use Cas6 for crRNA processing (Hale et al., 2009; Majumdar et al., 2015; Peng et al., 2015; Nickel et al., 2019; Wei et al., 2019). Structural analyses of different Cas6/Cas5d-RNA complexes revealed that many repeats formed characteristic stable stem-loop structures. Accordingly, Cas6/Cas5d enzymes have evolved distinct mechanisms to overcome the challenges of binding and catalysis of various RNA molecules (Figure 2; Koo et al., 2013; Li, 2014; Shao et al., 2016; Sefcikova et al., 2017).

Mature crRNA production has been reported for each subtype of type I. Repeats of CRISPR arrays from type I-E of *E. coli* and *Thermus thermophilus* and I-F of *Pseudomonas aeruginosa* were reported to form stable canonical stem-loop structures (Figures 2A–C; Haurwitz et al., 2010; Sashital et al., 2011; Sternberg et al., 2012). Interestingly, all of the stems and loops greatly vary in both size and sequences, which serve as bases for specific binding of Cas6 and subsequent cleavage. The Cas6 proteins specifically recognize and cleave the pre-crRNAs in a single turnover fashion, forming a stable hairpin structure in each repeat (Haurwitz et al., 2010; Sashital et al., 2011; Sternberg et al., 2012). After cleavage, they remained binding to the cleavage products by associating with the 3′ stem-loop structure tightly (Jore et al., 2011). This is consistent with the finding that Cas6 protein is an integrated part of the respective interference complexes (Brouns et al., 2008; Jore et al., 2011; Wiedenheft et al., 2011a, b; Jackson et al., 2014; Mulepati et al., 2014; Zhao et al., 2014). Similarly, repeats of type I-C from *Bacillus halodurans* also formed stable stem-loop structures, which, however, were produced by a unique Cas5 variant (Garside et al., 2012; Nam et al., 2012; Koo et al., 2013; Punetha et al., 2014) [known as Cas5d where the affix “d” refers to “Dvulg” (Haft et al., 2005)], as a *cas6* gene is missing from this system. A 3-bp stem and a tetra-loop included in the hairpin region were illuminated to be a minimal structural requirement for Cas5d recognition and cleavage (Nam et al., 2012).

Differently, CRISPR repeats present in type I-A of *Sulfolobus solfataricus* and type I-B of *Methanococcus maripaludis* usually form mini stem-loop structures (Figures 2D,E), instead of showing clear palindromic features (Kunin et al., 2007; Shao et al., 2016). For instance, in type I-A system, the formed stem-loop is composed of a 3-bp stem and a 5-nt loop, which were specifically recognized and stabilized by SsCas6, a Cas6 homolog. Two base pairs at the base of the stem were essentially required for SsCas6 binding and cleavage, as mutations disrupting the base-pair matching resulted in cleavage inhibition (Shao and Li, 2013). Likewise, a 3-bp stem could be potentially formed by the CRISPR repeats from another type I-B system of *Haloferax volcanii* (Maier et al., 2013), which likely contributes to the binding specificity of the HvCas6. Comparably, type I-G system shares the similar Cas6 homolog protein with coexisting types I-A and III-B systems in *Pyrococcus furiosus* to recognize CRISPR repeats with no obvious stem-loop structure for mature crRNA production (Figure 2F; Carte et al., 2010; Elmore et al., 2015).

crRNA processing in a type I-D system is the least known. Processing of CRISPR transcripts of a type I-D system in *Synechocystis* species PCC 6803 involved a Cas6 protein (Cas6-1) (Scholz et al., 2013; Jesser et al., 2019). Depletion of Cas6-1 from the cells largely decreased the crRNA amount, while this deficient phenotype could be restored through expression of Cas6-1 via a plasmid (Scholz et al., 2013). A mini stem-loop was also predicted to be formed in each repeat (Jesser et al., 2019), but whether it serves as the binding signal for Cas6-1 remains uncertain.

### Effector Machinery Assembly

Type I CRISPR-Cas systems encode multiple Cas proteins to multi-Cas effector machineries, which is termed the *CRISPR-associated complex for antiviral defense* (Cascade). Cascade was initially used for characterizing the type I-E CRISPR-Cas effector complex of *E. coli* (Brouns et al., 2008). This complex has a molecular weight of approximately 405 kDa and comprises proteins of Cas8e (also known as CasA or Cse1), Cas11 (also known as CasB or Cse2), Cas7 (also known as CasC or Cse4), Cas5 (also known as CasD), and Cas6e (also known as CasE or Cse3), as well as an RNA component, the 61-nt crRNA (Jore et al., 2011). These proteins shape a seahorse-like architecture with a stoichiometry of Cas8e_1_-Cas11_2_-Cas7_6_-Cas5_1_-Cas6e_1_ (Figure 1C; Jore et al., 2011).

**FIGURE 3:**
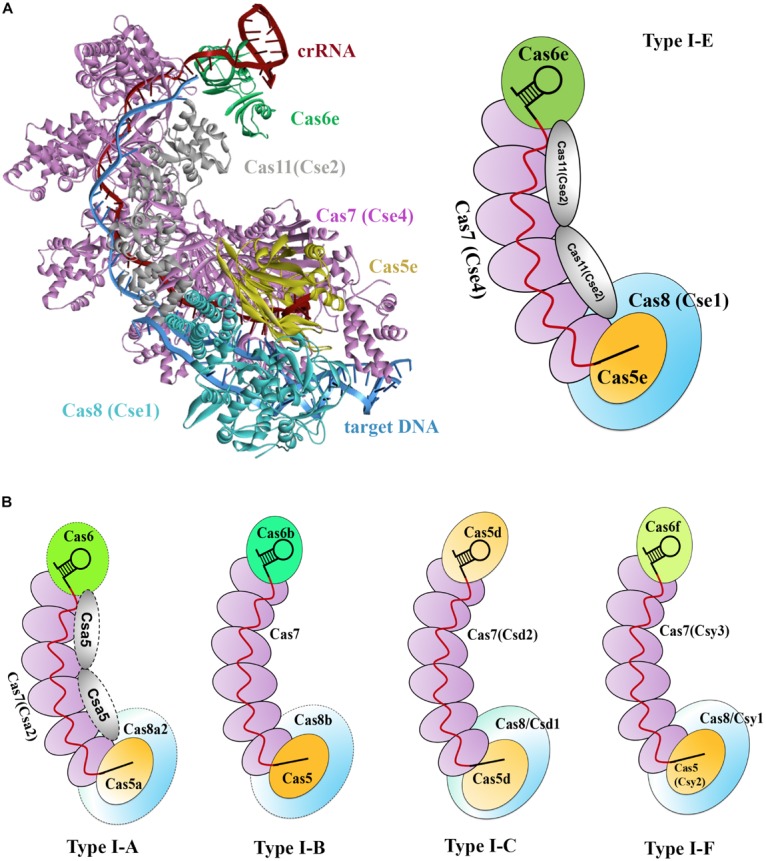
Models of type I Cascade complexes. **(A)** Structure of the type I-E Cascade of *E. coli* binding to a dsDNA target (PDB code: 5H9F, left) and a simulated model according to the structure (right). **(B)** Models for other characterized Cascade complexes of type I systems, including type I-A (Lintner et al., 2011), type I-B (Brendel et al., 2014), type I-C (Nam et al., 2012), and type I-F (Wiedenheft et al., 2011a), showing overall architectural similarities to that of the type I-E. Weakly associated subunits are indicated with dashed outline.

Two crystal structures of the type I-E Cascade bound to a crRNA have been solved, offering molecular details in the Cascade complex assembly (Figure 3A; Jackson et al., 2014; Zhao et al., 2014). In *E. coli*, as previously mentioned, pre-crRNA is processed by the Cas6e ribonuclease through cleavage of pre-crRNAs within each repeat. After processing, Cas6e remains tight binding to the 3′ repeat portion of each crRNA (Jore et al., 2011). At the same time, six Cas7 proteins run along the guide region (spacer) of the crRNA, forming the backbone filament, with the upper most subunit interacting with Cas6e. These Cas7 proteins adopt a right-hand-like shape (Mulepati et al., 2014), and the subunits are connected with one another by interaction between the “thumb” and “fingers” domains of one subunit and its adjacent subunits, respectively (Jackson et al., 2014). The Cas5e protein also exhibits a right-hand-like shape but lacks the “fingers” domain, thus allowing Cas5e to cap the Cas7 filament at the 5′ end of the crRNA by undertaking the same interaction of Cas7 with that between Cas7 subunits. In addition, Cas5e protein also holds the first 6 nt of the 5′ repeat portion of the crRNA (Jackson et al., 2014). In the seahorse-like complex, two Cas11 proteins form a dimer that does not directly interact with crRNA but functions as a bridge to connect the head and tail of the complex. One Cas11 subunit that is proximal to the 5′ end of crRNA, along with Cas5e, contacts Cas8 at the bottom of the complex, whereas the other Cas11 subunit interacts with Cas6e on the top (Jackson et al., 2014). Cas8 and Cas11 were identified as the largest and smallest subunits of the Cascade, respectively (Makarova et al., 2011a).

Subsequently, several other type I systems complexes were isolated and characterized (Lintner et al., 2011; Nam et al., 2012; Brendel et al., 2014). Comparison of the overall architectures of these complexes with that of the type I-E Cascade revealed that they share striking architectural similarities and thus being all referred as Cascade (Figure 3B; Reeks et al., 2013). These Cascade complexes show some common features. (1) A core complex of Cas5, Cas7, and/or Cas6 holds a crRNA, each protein possessing at least one RRM motif. (2) Cas7 subunits form the backbone of the complex and are more abundantly present. (3) The large subunit (Cas8) and/or small subunit (Cas11), if present, less tightly associate with the core complex. Thus far, a separate small subunit is only seen in type I-A and I-E systems, that is, Csa5 and Cse2, respectively. This small subunit is absent from the Cascade complexes of type I-B, I-C, and I-F, but the large subunits of these complexes are speculated to have contained a domain that is functionally homologous to the small subunit (Makarova et al., 2011a). Interestingly, the determined Cascade structure of a type I-F variant (I-Fv) lacks both the large and the small subunits, whose functions, however, are replaced by Cas5fv and Cas7fv, respectively (Pausch et al., 2017). The architectural similarities suggest that these Cascade complexes may use similar mechanisms for complex assembly and DNA interference.

### PAM Recognition and R-Loop Formation

The Cascade is directed to an invading DNA molecule solely relying on base pairing between the embedded crRNA and protospacer. An outstanding question would be how crRNA differentiates protospacer from the corresponding spacer that is stored in the genomic CRISPR bank. The evolved mechanisms for these systems to avoid self-CRISPR targeting involve a sequence immediately flanking the protospacer, called a protospacer adjacent motif (PAM), which is essentially required for Cascade to determine *bona fide* DNA targets. First predicted by Mojica et al. (2009) and experimentally demonstrated for type I-A systems in *Sulfolobus* by Gudbergsdottir et al. (2011), PAM is typically of two to five base pairs and located at the 5′ end of protospacer on the strand matching the spacer.

Protospacer adjacent motif recognition has also been studied in other type I systems. In type I-E system of *E. coli*, a loop structure, named the L1 loop that is located within the N-terminal domain of Cas8, directly contacts with the PAM, mediating specific binding of Cascade to the PAM-containing DNA target (Sashital et al., 2012). Further analyses defined that three structural features, including a glutamine wedge, a glycine loop, and a lysine finger, were required for PAM recognition by Cas8 and specified the interaction of PAMs with the target strand (Hayes et al., 2016). In type I-E Cascade of *Thermobifida fusca*, the Cas8 subunit played the same roles in specifying the PAMs, while through contacting the non-target strand (Xiao et al., 2017). In both cases, PAM sequences were recognized by the Cas8 proteins at the minor groove side, explaining the promiscuity of PAM recognition in these systems. Additionally, the recognition of PAM by Cas8 homologs was also identified in type I-B systems of *H. volcanii* and *Methanothermobacter thermautotrophicus* (Cass et al., 2015). Interestingly, in the reported type I-Fv system, the Cas8 protein is missing. Instead, the existing Cas5f variant (Cas5fv) containing an additional domain may compensate for the roles of Cas8 (Pausch et al., 2017).

*Bona fide* PAM recognition and base pairing between a crRNA and the cognate protospacer determine a target DNA. Interestingly, in some initial work, researchers found that a few type I CRISPR-Cas systems exhibited tolerance to mismatches between the crRNAs and corresponding protospacers. Impacts of mismatches at different regions of the crRNA on target DNA interference markedly varied. In one of the studies, Wiedenheft et al. (2011b) used isothermal titration calorimetry to investigate DNA-binding affinity of the *P. aeruginosa* type I-F system. The results indicated that an 8-nt ssDNA oligo matching the first 8-nt 5′ guide sequence of a crRNA (1–8 nt) showed a high binding affinity of the crRNA, whereas another 8-nt ssDNA oligo complementary to the corresponding 5 to 12 region of the crRNA presented a 4-fold weaker binding affinity. Strikingly, all other tested 8-nt ssDNA oligos that matched the crRNA at a region outside the 1- to 8-nt stretch exhibited no measurable binding affinity. These results indicated that the 1- to 8-nt sequence within the crRNA, immediately adjacent to the PAM, played an essential role in defining an invading DNA as an attacker. This is in analogy to the seed sequence in small RNAs, which functions in target recognition in the RNAi in eukaryotes (Lewis et al., 2003).

Following binding of Cascade to a DNA target upon PAM recognition is the formation of a full R-loop, which was observed in several type I systems. Cascade binding could destabilize the target DNA duplex, allowing crRNA to first pair with the protospacer within the seed region and then throughout the whole matching sequences and thus forming a full R-loop, where the non-target strand is bound by the Cas11 dimer (Hochstrasser et al., 2014; Szczelkun et al., 2014). The existence of an intermediate seed bubble immediately following the PAM recognition but before the full R-loop formation was recently evidenced. Xiao et al. (2017) captured a structural snapshot of the *T. fusca* type I-E Cascade, showing an 11-bp unwound sequence in the seed region. R-loop formation is accompanied by conformational changes of the small and large subunits that trigger recruitment of Cas3 for target degradation (Hochstrasser et al., 2014).

### DNA Target Degradation

As aforementioned, Cascade recruited Cas3 to unwind, cut, and degrade the targets. Cas3 typically comprises an N-terminal HD phosphohydrolase domain and a C-terminal superfamily 2 helicase domain (Makarova et al., 2006). There are also exceptional cases in some type I systems. For example, these two domains are encoded as individual proteins, Cas3′ (helicase) and Cas3″ (nuclease), respectively, in type I-A of *S. islandicus* Rey15A (Figure 1B; Guo et al., 2011; Makarova et al., 2011b). It was speculated that the HD domain carried out the divalent ion-dependent catalytic cleavage on ssDNA and/or RNA (Han and Krauss, 2009; Beloglazova et al., 2011; Mulepati and Bailey, 2011; Sinkunas et al., 2011), whereas the helicase domain unwound the DNA/DNA and/or DNA/RNA duplexes in the presence of ATP (Howard et al., 2011; Sinkunas et al., 2011).

The mechanism of target DNA degradation by Cas3 was revealed by structural analyses of type I-E targeting complexes, in which the recruitment of Cas3 strictly required the formation of a full R-loop structure. The ape Cascade and R-loop-forming Cascade present as different conformers and Cas3 only selectively capture the latter (Xiao et al., 2017). This conformational difference might act as signals for triggering Cas3 recruitment and also help the type I systems avoid cleaving partially complementary sequences (off-targeting). Upon the formation of Cas3-Cascade-DNA target complex (Huo et al., 2014), Cas3 nuclease nicked the non-target strand in the 7- to 11-nt region of a protospacer (Hochstrasser et al., 2014). The non-target strand contained a significant bulge structure in the R-loop that was created by the Cascade complex, facilitating its handover from Cascade to the bound Cas3 for nicking (Xiao et al., 2017). This handover was recently confirmed to be essential for type I-E immunity (Xiao et al., 2018). Subsequent exonucleolytic degradation of the DNA target was observed to occur in the direction of 3′ to 5′ with the need of ATP. Sinkunas et al. (2013) found that Cas3 cleaved only on the displaced strand in the R-loop in absence of ATP, but destroyed both strands when ATP was present. This may indicate that unwinding DNA target by the ATP-dependent Cas3 helicase domain could further provide ssDNA substrates for the nuclease domain of Cas3, or for other host nucleases, leading to degradation of the entire DNA target (Brouns et al., 2008; Mulepati and Bailey, 2013).

### A Deduced General Mechanism of Type I DNA Interference

**FIGURE 4:**
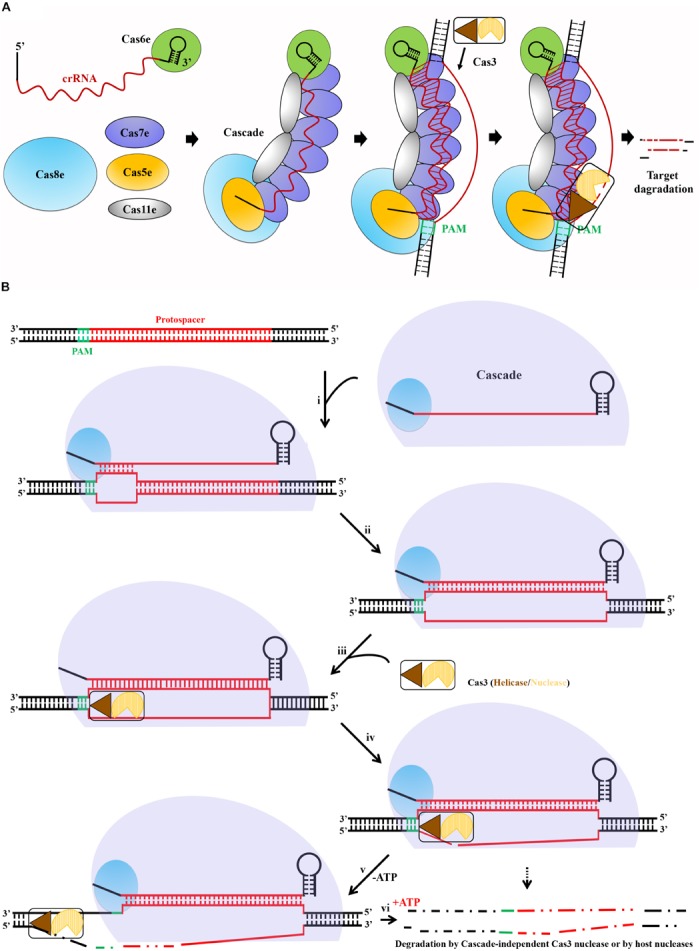
A deduced general mechanism of type I Cascade-mediated DNA interference. **(A)** Schematic showing interference pathway exemplified by the type I-E CRISPR-Cas system of *E. coli*. crRNA produced and bound by Cas6e acts as a scaffold for Cascade assembly. Upon recognition of PAM (green) on the invading DNA, Cascade bound to the target DNA, followed by R-loop formation after crRNA base pairs with the target strand DNA, triggering the recruitment of the endonuclease Cas3. Finally, Cas3 initials degradation of the non-target strand. **(B)** The crRNA and type I Cas proteins form a Cascade complex. If present, an optimal PAM in a DNA target, Cas8 interacts directly with the PAM, allowing Cascade binding. A primary base pairing between the crRNA and protospacer within the seed (i) is followed by extended base pairing, displacing the non-target strand and forming a full R-loop (ii). Conformational changes caused by target DNA binding trigger Cas3 helicase/nuclease to join in the complex, docking at a Cas8-provided site (iii). In the absence of ATP (-ATP), the nuclease domain cuts the displaced strand within the protospacer (iv), leaving a ssDNA gap in the target (v). In the presence of ATP (+ATP), Cas3 helicase unwinds the dsDNA, and complete degradation of the target DNA is mediated by either the Cascade-independent Cas3 nuclease activity or other host nucleases (vi).

Collectively, as exemplified for the type I-E system in Figure 4A, all known type I CRISPR-Cas systems form a Cascade machinery to perform DNA interference in several steps, consisting of multiple protein components complexed with a mature crRNA molecule. A general mechanism of type I Cascade-mediated DNA interference could be deduced as described following and shown in Figure 4B. The interference begins with the recognition of a suitable PAM by Cas8. Direct interaction between the L1 loop of Cas8 and the PAM (Hochstrasser et al., 2014) locates the Cascade to the DNA target and destabilizes the DNA duplex (Szczelkun et al., 2014). The crRNA base pairs with the protospacer, first within the seed region and then throughout the whole matching sequences, to eventually displace the non-target strand, forming a full R-loop. On the other hand, the initial dsDNA binding induces a major conformational change to the Cascade (Wiedenheft et al., 2011a), which may trigger the recruitment of Cas3 to the Cas8 docking site (Westra et al., 2012; Hochstrasser et al., 2014). It was reported that once the non-target strand is displaced by the crRNA, it is exposed and handed over to the Cas3 nuclease for nicking and successive degrading in the 3′ to 5′ direction (Sinkunas et al., 2013; Gong et al., 2014). This reaction may generate an intermediate degradation product, as partially ssDNA might not result in complete target DNA degradation. In fact, complete degradation of the target DNA is probably mediated either by the nuclease domain of Cas3 or by other host nucleases with assistance of the ATP-dependent helicase domain of Cas3 to unwind the DNA target (Brouns et al., 2008; Mulepati and Bailey, 2013).

## Practical Applications of Type I CRISPR-Cas Systems

**FIGURE 5:**
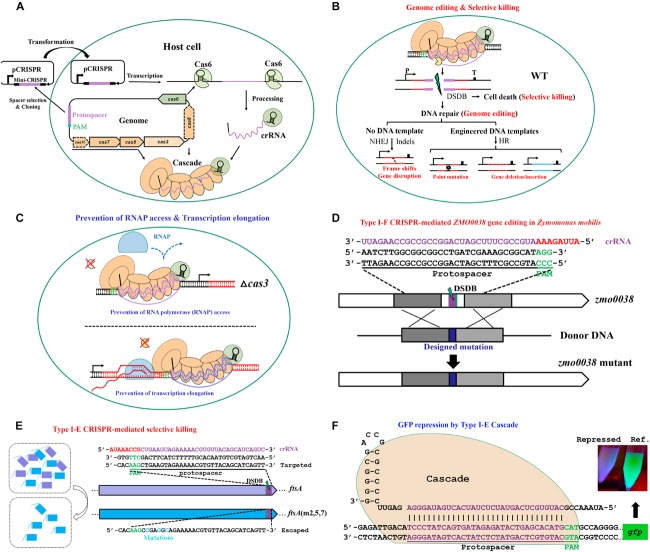
The basic working principle of type I Cascade-based technologies. **(A)** Strategy for native type I Cascade-based applications. A DNA stretch immediately 5′ downstream of a PAM is selected for mini-CRISPR construction. crRNA expressed from the plasmid-borne mini-CRISPR array forms the Cascade complex with Cas proteins expressed from the genomic *cas* operon. **(B)** Architecture of type I Cascade-mediated genome engineering. **(C)** Architecture of type I Cascade-mediated gene expression modulation. The Cascade is directed to the target site in the host genome, either recruiting the Cas3 to generate a double-stranded DNA break (DSDB) in wild-type (WT) cells **(B)** or binding to the target tightly without cleaving it in a *cas3* knockout background **(C)**. **(D)** Schematic showing an example of genome editing by repurposing the type I-F of *Zymomonas mobilis* [constructed according to Zheng et al. (2019)]. **(E)** Schematic showing an example of selective killing by using the type I-E of *E. coli* [constructed according to Gomaa et al. (2014)]. **(F)** Schematic showing an example of gene expression control by the type I-E Cascade in *E. coli* [modified according to Rath et al. (2015)].

The above mechanistic dissections of type I CRISPR-Cas–based immunity have provided solid theoretical basis for exploiting these systems for a wide range of practical applications in bacterial and archaeal hosts. By simply designing a plasmid-borne artificial mini-CRISPR to express genome-targeting crRNAs, a type I Cascade complex can be readily directed to a genome sequence to accomplish various tasks (Figure 5A). These applications can be briefly classified into two classes according to the different activities of these systems. One utilizes the intact DNA interference function of Cascade and Cas3 (Figure 5B), for example, genome editing, natural variants selection, and antimicrobials, and the other uses only the target surveillance and binding ability of Cascade alone (without Cas3 nuclease) (Figure 5C), for example, gene expression regulation.

### Genome Editing

To date, genome editing predominates over other CRISPR applications and remains the best developed, however, overwhelmingly focusing on eukaryotes. In the eukaryotic organisms, double-stranded DNA breaks (DSDBs) introduced by CRISPR-Cas systems at specific locations of the genome could be repaired through either the cellular non-homologous end jointing (NHEJ) or the homologous recombination (HR) repair pathways. The NHEJ repair system functions in an error-prone manner that usually generates indels (insertions/deletions) within the vicinity of the target site, leading to frame shifts or gene disruption. In contrast, HR repair system normally creates precise changes at desired positions in the genome when providing engineered DNA templates. Dissimilarly, most prokaryotic organisms harbor only the HR system. It has to be pointed out that although a bacterial version of NHEJ, involving two proteins, Ku and LigD, was reported (Weller et al., 2002), it occurs only in a very small portion of bacteria. In general, prokaryotes repair the CRISPR-caused DSDBs with relatively much lower efficiencies, which is consistent with the frequently observed CRISPR-mediated lethality when self-targeting crRNAs of active systems were introduced into the prokaryotic cells (Edgar and Qimron, 2010; Vercoe et al., 2013; Gomaa et al., 2014; Peng et al., 2015). It is reasonable because the invaders would persist to affect the host cells if the cleavage of invading attackers, such as bacteriophages and conjugative plasmids, by Cas nucleases is subsequently repaired. In practical applications, this is the main obstacle that needs to be addressed in harnessing the endogenous type I systems for genome editing.

Via coupling the DNA targeting activity of endogenous type I CRISPR-Cas systems with the DNA-assisted HR mechanism, various genome editing purposes can be achieved, including gene insertion, deletion, replacement, tagging, and nucleotide substitution, as well as multiplexed gene deletion, in their natural hosts. Here, we take our recently published work on type I-F CRISPR-Cas system as an example to introduce the procedure, strategy, and principle of exploiting an endogenous type I system for genome editing in *Zymomonas mobilis* (Zheng et al., 2019). In the study, we first demonstrated the DNA cleavage activity of the endogenous type I-F CRISPR-Cas of *Z. mobilis* and determined the PAM sequences essential for the system through a plasmid invader assay (Peng et al., 2013, 2015). This activity was then directed to genomic locations by crRNAs produced from plasmid-borne artificial CRISPR loci to generate DNA injuries. In addition, donor DNAs were supplied as inserts of the same plasmids for facilitating HR, allowing their multiplication via plasmid replication to ensure effective concentration and stability, which has been evidenced to be an efficient approach for enhancing the HR rate and hence genome editing efficiency (Li et al., 2016; Renaud et al., 2016; Cheng et al., 2017). Using this established type I-F CRISPR-Cas platform, various genome engineering purposes were efficiently achieved with efficiencies of up to 100%, including gene deletion, replacement, *in situ* modifications, particularly large genomic fragment deletion, and simultaneous removal of three genes (Figure 5D). Moreover, our work demonstrated that depletion of a DNA restriction-modification (R-M) system could also lead to boosted genome engineering efficiency (Zheng et al., 2019). Considering the widespread of R-M systems (Koonin et al., 2017a) and other DNA interference systems (Doron et al., 2018) in microorganisms, the method could be served as an important reference for the development and deployment of similar CRISPR-Cas toolkits in organisms with low efficiency in the wild-type genetic background.

Endogenous type I CRISPR-based genome editing was first reported in *S. islandicus*, and then other organisms. Gene deletion and site-directed mutagenesis were completed in the Crenarchaeota *S. islandicus* by constitutively expressing its native type I-A system (Peng et al., 2013; Li et al., 2016). After transforming a plasmid bearing both the CRISPR target and donor DNA template into the host cells, almost all the analyzed transformants were found to possess precise changes including deletions and multiple point substitutions as designed (Li et al., 2016). Likewise, the endogenous type I-B system of the halophilic archaeon *Haloarcula hispanica* was also redirected for gene deletion, insertion, point mutations introduction, and simultaneous deletion of two genes (Cheng et al., 2017). Besides, two other type I-B systems from the bacteria *Clostridium tyrobutyricum* and *Clostridium pasteurianum*, respectively, have also been used for genome editing in their native hosts, with an editing efficiency of 100% on gene deletion/insertion (Pyne et al., 2016; Zhang et al., 2018). Strikingly, the native type I-B system of *C. pasteurianum* facilitated more efficient genome editing of around 4-fold higher than that of the heterologously expressed Cas9 system (Pyne et al., 2016). Very recently, the type I-E system of *Lactobacillus crispatus* was also exploited to perform *in situ* genomic modifications in the host cells (Hidalgo-Cantabrana et al., 2019b).

Targeted DNA integration can also be accomplished using crRNA-guided transposition, which was first proposed by Peters et al. (2017). It was found that a number of CRISPR-Cas systems are carried by transposons belonging to the Tn7 family (Peters, 2014), and they possess a notable feature of lacking a key factor responsible for DNA targeting. Moreover, the CRISPR-Cas systems carried by them are not capable for DNA interference. This proposed mechanism has been recently experimentally confirmed in *E. coli* by two groups (Klompe et al., 2019; Strecker et al., 2019). In one instance, Klompe et al. (2019) show that a variant type I-F system from *Vibrio cholerae*, where the adaptation module and Cas3 nuclease are missing, could still form a Cascade complex and guide transposition into specific sites 46 to 55 bp downstream of crRNA-matching sequences. Interestingly, the transposition can be done only with the coevolved type I-F variant, as other tested native CRISPR-Cas systems did not work properly (Klompe et al., 2019). Moreover, the authors also found that the Cascade could be copurified with a TniQ protein, an element of the Tn7 system involved in one of the characterized Tn7 transposition pathways (Peters, 2014). More interestingly, this crRNA-guided transposition could be observed only when expressing a fusion of TniQ-Cas6, but not TniQ-Cas8. These observations thus allowed the authors to propose a mechanism that the CRISPR-Cas system could cofunction with the Tn7 system (Klompe et al., 2019). Similarly, Strecker et al. (2019) reported that the Tn7-like system could recruit a type V-K variant from *Scytonema hofmannii*. This mechanism was later further supported by a cryo-EM structure of TniQ-Cascade complex (Halpin-Healy et al., 2019), within which two TniQ subunits form a dimer, with each interacting with Cas6 and the immediately adjacent Cas7 subunit, respectively. Finally, with either system, CRISPR-directed DNA insertion has been attained in a programmable fashion with high frequencies. More importantly, with no requirement for target DNA cleavage, homologous DNA templates, endogenous DNA repair systems, and selective pressure, this strategy is advantageous for genome modification over many other existing tools.

### Selective Killing

As aforementioned, reprogramming an active Cascade toward the chromosome without providing a repair template would lead to killing of most, if not all, targeted cells. Two alternative fates might be brought to the targeted cells, including the death of wild-type cells due to CRISPR-mediated chromosomal degradation, or the survival if variants carry mutations in the targeted sequences. This selective killing feature can be adopted to exploit DNA-cleaving CRISPR-Cas systems as screening tools to select for genetic variants from a given population or as novel programmable antimicrobials to selectively remove certain pathogens from a mixed population (Figure 5B).

In an early study, Edgar and Qimron (2010) demonstrated that the native type I-E CRISPR-Cas system of *E. coli* could be used to cure the cells from prophage. The induced endogenous system could lead to killing of more than 98% of the cells in the population when the targeting activity was directed to an integrated lambda prophage. Interestingly, although prophage induction was also lethal to the cells, simultaneously inducing both of the pathways largely enhanced the survival rate. Further analyses showed that the genome of survivors exclusively did not harbor any prophage sequences. According to the observations, the authors suggested that the survived cells were possibly protected by the CRISPR system acting on excised bacteriophages, and most cells that still harbored prophage genes in the genome were killed by DNA targeting (Edgar and Qimron, 2010). Later, self-targeting by a native type I-F system was reported to result in dramatic changes in the host’s genome (Vercoe et al., 2013). Either part of or an entire preexisting pathogenicity island was deleted from the chromosome when targeted by the type I-F CRISPR-Cas system of *Pectobacterium atrosepticum*, a potato phytopathogen. The chromosomal alterations allowed the genetic variants to survive from CRISPR targeting and resulted in strains lacking pathogenicity, which was proposed to be a strong selective pressure for bacterial evolution (Vercoe et al., 2013).

The sequence specificity and selective killing feature of CRISPR-mediated genome targeting have enabled its antimicrobial utilization (Figure 5E), a concept that was put forward by Gomaa et al. (2014). Gomaa and coworkers assessed the genome targeting efficiency of the native type I-E CRISPR-Cas system from *E. coli* through evaluating cell escape rates. It was found that targeting single or multiple sites within coding or non-coding region of essential or non-essential genes on either strand of the genome gave similar outcomes of a dramatically low cell escape rate, demonstrating that the targeting was efficient and specific yet flexible, requiring only the target sequence with an optimal PAM. This specificity and flexibility allowed selective removal of individual or multiple strains, including highly similar ones, from pure or mixed cultures by directing the targeting activity to a unique or shared PAM-flanking genomic sequence (Gomaa et al., 2014). The application of type I-E CRISPR-Cas system of *E. coli* as antimicrobials was echoed in another study, in which Yosef et al. (2015) engineered a lambda prophage to deliver an active CRISPR-Cas system along with an artificial CRISPR that expresses crRNAs targeting antibiotic resistance genes into the *E. coli* host cells. After the delivery, the lysogenized *E. coli* cells were immunized against the corresponding antibiotic resistance gene-housing genetic elements, not only clearing the preexisting resistance determinants to sensitize the antibiotic-resistant bacteria, but also blocking further uptake of the same resistance. Importantly, the sensitized cells were protected by the delivered CRISPR-Cas system against lytic bacteriophages bearing the same targeting sequences, whereas the residual antibiotic-resistant cells would be persistently attacked by such type of bacteriophages, thus bringing out obvious selective benefit to the sensitized cells over the resistant ones. In addition, two endogenous type II CRISPR-Cas systems of *Streptococcus thermophilus* were also proven to be able to selectively remove closely related organisms by targeting unique sequences from a mixed population of microbes (Gomaa et al., 2014). Therefore, potent selective killing can be gained through different endogenous CRISPR-Cas systems. In fact, if provided with an appropriate delivery vehicle, heterologous CRISPR-Cas can be used as a programmable antimicrobial as well. In two parallel works, a heterologous type II CRISPR-Cas system was delivered into *E. coli* by an M13-based phagemid (Citorik et al., 2014) and into *Staphylococcus aureus* by a ΦNM1-based phagemid (Bikard et al., 2014), respectively, to selectively killing the pathogenic host cells.

In all above cases, both types I and II CRISPR self-targeting could give very robust selective pressure, thus obscuring the difference of targeting effect between these two types of CRISPR-Cas. As illustrated in Figure 4B, Cas3 in type I is mainly responsible for target destruction, first nicking and then degrading DNA via its 3′- to -5″ exonuclease activity. In contrast, Cas9 in type II only cuts the target DNA. It would be interesting to evaluate whether DNA degradation contributes to further increased potency of killing through DNA repair prevention. Nevertheless, these CRISPR-based antimicrobials will undoubtedly limit the increasing threat of antimicrobial resistance microorganisms to the global public health.

### Gene Expression Modulation

Type I CRISPR-Cas systems use the protein elements of Cascade and Cas3 for DNA interference. Depletion of Cas3 from a native type I system would still allow Cascade to tightly bind to the target DNA but without cleaving it. Thereby, the standalone Cascade becomes a transcriptional barrier to prevent either RNA polymerase access or transcription elongation, thus being capable of gene expression regulation (Figure 5C).

The capability of type I CRISPR-Cas systems in transcriptional regulation has been demonstrated for type I-E system of *E. coli* (Figure 5F) in two parallel studies, in both of which the Cas3 nuclease was eliminated (Luo et al., 2015; Rath et al., 2015). In *E. coli*, expression of the Cascade operon is largely repressed under laboratory conditions (Pul et al., 2010; Westra et al., 2010). In order to address this issue, Luo et al. (2015) generated a mutant strain, in which the transcription of Cascade operon was modified to be driven by a constitutive promoter, and Rath et al. (2015) exogenously introduced an extra copy of Cascade operon via a plasmid. These strains kept the ability to process the plasmid-borne CRISPR arrays containing either a single spacer or multiple spacers. As expected, the produced crRNA guided the Cascade to the target sites and exhibited strong repression effect on expression of a genome-integrated *gfp* reporter gene, especially when targeting the promoter region. Moreover, simultaneously repressing several genes was also assayed to be effective by taking the advantage that multiple unique mature crRNA molecules could be produced from a single CRISPR array (Luo et al., 2015; Rath et al., 2015).

Stachler and Marchfelder (2016) similarly, used the endogenous type I-B CRISPR-Cas system to perform *in vivo* gene down-regulation in the archaeon *H. volcanii*. Also, the *cas3* gene was either deleted or mutated to disable its catalytic activity to avoid genome cleavage. In addition, the crRNA-producing Cas6 protein was further depleted from the system, such that the genome-encoded CRISPR arrays would not be processed. Instead, the targeting crRNAs were encoded from a plasmid (Stachler and Marchfelder, 2016) and processed into mature crRNAs via a Cas6-independent approach for *H. volcanii* (Maier et al., 2015). This was evidenced to be significant for the interference efficiency, suggestive of competition between the targeting crRNA and internal crRNAs for Cascade and pointing toward the fact that Cascade is a limiting factor of type I silencing in this strain. Based on the strain modifications, efficient gene repression was seen, with the greatest reducing of transcripts by 92% when targeting the promoter region. Strand bias was also observed that Cascade targeting to the template strand showed much higher silencing efficiency than targeting to the non-template strand (Stachler and Marchfelder, 2016). In our recent work, gene expression repression by an endogenous type I-F system was also successfully achieved for *Z. mobilis* (Zheng et al., 2019).

Aside from repression of independent genes, type I CRISPR interference was coopted for pathway engineering as well. In a pioneering work, the endogenous type I-E system was used to regulate catabolism of four sugars by targeting their respective operons in *E. coli*. All the four targeted sugar catabolism pathways were efficiently silenced by simply expressing a four-spacer CRISPR array (Luo et al., 2015). Subsequently, using the same endogenous system targeting *gltA* gene in *E. coli* also highly repressed the citrate synthase, leading to accumulation of acetate and thus regulation of the metabolic flux of central metabolism (Chang et al., 2016). Recently, Tarasava et al. (2018) applied CRISPR interference strategy to simultaneously alter the expression levels of multiple genes associated with 3-hydroxypropionate (3-PH) production and finally achieved nearly 2-fold increase of 3-PH yield in *E. coli*. In order to controllably utilize the type I-E system for CRISPR interference, the authors replaced the native promoter of Cascade operon with an arabinose-inducible promoter in a *cas3* deletion mutant. Considering that the placement of spacers within the cluster would influence the repression strength (Luo et al., 2015), the effect of different orders of spacer combination on the enhancement of 3-PH production was tested. On the other hand, a combinatorial library of gRNA array targeting six genes was built and subsequently used to screen for highest producing variants in this work, which was estimated to contain approximately 10^4^ unique variants and approximately three orders of magnitude more than that analyzed in the rational design approaches (Lv et al., 2015; Wu et al., 2015). Importantly, this library was proven to be diverse combinatorial as 48 out of 50 sequenced variants were found to be unique to each other. Furthermore, the orders of spacer combination harbored by some outstanding producing variants screened from the library were highly consistent with the rationally engineered variants (Tarasava et al., 2018).

All these studies have paved the way for further development of type I CRISPR-Cas-based *in vivo* gene interference tools in bacteria and archaea. Considering the broad occurrence of diverse type I systems, it is of great interest to see whether other subtypes are able to perform gene silencing in many other bacteria and archaea.

## Conclusion and Perspectives

Based on the extensive studies on characterizing the type I CRISPR-Cas functions, a general mechanism of type I DNA interference can be deduced (Figure 4B). The functional demonstrations have contributed great efforts for exploiting the endogenous type I systems as CRISPR toolkits for genome editing, antimicrobials, gene regulation, and so on, in bacteria and archaea. While many successful achievements have already proven type I CRISPR-based technologies to be powerful tools for prokaryotic engineering, they are still in their infancy stage, leaving many hurdles for their deeper improvements for a wider range of applications.

### Hurdles Ahead

To truly exploit the endogenous type I CRISPR-Cas systems, there are several critical obstacles still required to be addressed. First, because of the host-specific property of endogenous CRISPR-Cas systems, each of them must be thoroughly characterized in the host cells, including determination of PAM sequences, demonstration of Cas functions, and identification of mature crRNAs, as well as dissection of its mode of action during foreign DNA defense. Second, low efficiency of exogenous DNA delivery and their low stability in host cells might significantly hamper the repurposing of endogenous CRISPR-Cas. Although there is no need of importing exogenous Cas proteins, other genetic materials, for example, artificial CRISPR arrays, and donor DNA templates during genome editing, are still required to be introduced into the cells with relatively high concentrations. Therefore, efficient delivery systems would help for better performances during the applications. Separately, prokaryotes harbor a collection of innate defense systems, such as R-M (Roberts et al., 2015), BREX (Goldfarb et al., 2015), and so on. Many of these systems are prevalent; for example, approximately 95% of sequenced bacterial genomes encode at least one R-M system (Roberts et al., 2015). Research has shown that destroying such innate defense systems can lead to boosted plasmid transformation rate and hence genome engineering efficiency (Zheng et al., 2019). In addition, because most of prokaryotes use the less efficient HR pathway for repairing the CRISPR-generated DNA injuries, it is always good to maintain a high concentration of introduced donor DNAs to ensure a better performance of genome editing. To this end, several strategies can be used, such as supplying DNA templates as inserts of the vectors to allow their multiplication (Li et al., 2016; Cheng et al., 2017; Zheng et al., 2019). Finally, during the CRISPR-targeting, escape always occurs with various modes, normally due to modifications/alterations in spacer or protospacer, or alternatively mutations in Cas proteins. However, it would be important but troublesome to understand the escape modes and underlying mechanisms, which might provide possibility for further increasing the overall efficiency of genome manipulations.

### CRISPR-dCas3 Exploitation

Current applications of type I CRISPR-Cas systems focus mainly on genome editing and gene down-regulation. For the latter, in most studies, the *cas3* gene was deleted from the system. Actually, a system with a catalytically inactive Cas3 (dCas3) would have further uses. For instance, considering DSDBs are repaired with relatively lower efficiencies in prokaryotic cells, it would be beneficial to use a catalytically inactive Cas protein, for example, dCas9 or dCas3, with the *Fok*I endonuclease to generate DSDBs in a manner that can be more easily repaired by the endogenous pathways, hence increasing engineering efficiency (Guilinger et al., 2014; Tsai et al., 2014). In an even efficient manner, single DNA changes within a targeted window can be achieved without introducing DSDBs by using deaminase–dCas9 fusions in *E. coli* (Gaudelli et al., 2017; Banno et al., 2018), where the CRISPR-dCas9 system might be potentially replaced with an endogenous CRISPR-dCas3 system. Additionally, dCas3, together with the Cascade, may enrich a fusing transcription activator to a genomic region and may thus lead to activation of targeted genes, just in parallel with the similar applications of dCas9/dCas12 as reviewed by Yao et al. (2018). In short, attempts of other applications through the endogenous type I CRISPR-dCas3 systems, such as cell imaging that has not been reported in prokaryotes, can be achievable and will further extend the application scope of CRISPR-based technologies.

## Author Contributions

WP conceived the outline with inputs from all authors. YZ and WP prepared and wrote the manuscript. WP and LY revised the manuscript. JL, BW, JH, YH, SW, XM, SY, and LM conducted the extensive review. All authors contributed to the writing, reading, and approval of the final manuscript.

## Conflict of Interest

The authors declare that the research was conducted in the absence of any commercial or financial relationships that could be construed as a potential conflict of interest.
